# Study Protocol on Ecological Momentary Assessment of Health-Related Quality of Life Using a Smartphone Application

**DOI:** 10.3389/fpsyg.2016.01086

**Published:** 2016-07-18

**Authors:** Silvana Mareva, David Thomson, Pietro Marenco, Víctor Estal Muñoz, Caroline V. Ott, Barbara Schmidt, Tobias Wingen, Angelos P. Kassianos

**Affiliations:** ^1^Department of Psychology, University of EdinburghEdinburgh, UK; ^2^School of Psychology, University of GlasgowGlasgow, UK; ^3^Department of Psychology, University of BolognaBologna, Italy; ^4^Department of Personality, Evaluation and Psychological Treatment, Faculty of Psychology, Autonomous University of MadridMadrid, Spain; ^5^Department of Psychology, University of CopenhagenCopenhagen, Denmark; ^6^Department of Ergonomics and Psychology, Budapest University of Technology and EconomicsBudapest, Hungary; ^7^Department of Psychology, University of CologneCologne, Germany; ^8^Department of Applied Health Research, University College LondonLondon, UK

**Keywords:** mobile health, health-related quality of life, ecological momentary assessment, sleep quality, real-time assessment, smartphone application

## Abstract

Health-Related Quality of Life (HRQoL) is a construct of increasing importance in modern healthcare, and has typically been assessed using retrospective instruments. While such measures have been shown to have predictive utility for clinical outcomes, several cognitive biases associated with human recall and current mood state may undermine their validity and reliability. Retrospective tools can be further criticized for their lack of ecology, as individuals are usually assessed in less natural settings such as hospitals and health centers, and may be obliged to spend time and money traveling to receive assessment. Ecological momentary assessment (EMA) is an alternative, as mobile assessment using mobile health (mHealth) technology has the potential to minimize biases and overcome many of these limitations. Employing an EMA methodology, we will use a smartphone application to collect data on real-time HRQoL, with an adapted version of the widely used WHOQOL-BREF questionnaire. We aim to recruit a total of 450 healthy participants. Participants will be prompted by the application to report their real-time HRQoL over 2 weeks together with information on mood and current activities. At the end of 2 weeks, they will complete a retrospective assessment of their HRQoL and they will provide information about their sleep quality and perceived stress. The psychometric properties of real-time HRQoL will be assessed, including analysis of the factorial structure, reliability and validity of the measure, and compared with retrospective HRQoL responses for the same 2-week testing period. Further, we aim to identify factors associated with real-time HRQoL (e.g., mood, activities), the feasibility of the application, and within- and between-person variability in real-time HRQoL. We expect real-time HRQoL to have adequate validity and reliability, and positive responses on the feasibility of using a smartphone application for routine HRQoL assessment. The direct comparison of real-time and retrospective measures in this study will provide important novel insight into the efficacy of mHealth applications for HRQoL assessment. If shown to be valid, reliable and feasible for the collection of HRQoL data, mHealth applications may have future potential for facilitating clinical assessment, patient-physician communication, and monitoring individual HRQoL over course of treatment.

## Introduction

Health-Related Quality of Life (HRQoL) constitutes a multidimensional construct for the interpretation of health states of individuals or groups. Health-Related Quality of Life explains variation in survival of chronic conditions such as cancer (Steel et al., 2014) and is associated with outcomes in non-clinical populations, such as better sleep quality (Ratcliff et al., 2014), activity levels (Bize et al., 2007) and exercise capacity (Lindholm et al., 2003). Further, routine assessment of HRQoL has been shown to improve patient-physician communication (Velikova et al., 2004).

The increasing importance of measuring HRQoL, particularly in clinical settings (Catania et al., 2015), has precipitated greater demand for the development of standardized measurement tools. Typically HRQoL is assessed using retrospective self-reports, which rely on participants’ ability to recall information from episodic memory. As episodic memory declines over time, individuals develop greater reliance on semantic memory to complete the resultant ‘gaps’ in recall (Maes et al., 2015). Constructive mental processes recombine elements of past events, and are prone to cognitive biases (Schacter, 2012). Specifically, when individuals respond to questions regarding their HRQoL, they estimate the intensity and frequency of experiences based on a set of highly subjective heuristics (Solhan et al., 2009).

Several cognitive biases compromise the validity of retrospective HRQoL assessment. Recall bias creates inaccuracies during retrospective assessment (Blome and Augustin, 2015) and undermines the statistical power and validity of HRQoL tools (Schwartz et al., 2004). The peak-end phenomenon is another cognitive bias involving the tendency to recall the most extreme and recent instances of an experience or feeling. The mood congruency effect refers to the employment of personalized heuristics to reconstruct memories. Therefore, individuals often use their current mood as a reference point rather than accurately recalling specific instances of moods (Solhan et al., 2009), resulting in better recall for states congruent with current mood, and potentially generating recall bias. Further, individuals with greater fluctuations in momentary experiences (e.g., pain, mood) recall instances less accurately than individuals with more stable feelings, upon weekly retrospective assessment (Stone et al., 2005).

The limitations of retrospective assessment necessitate the development of more robust tools. Modern advances in mobile health (mHealth) have facilitated ecological momentary assessment (EMA), the repeated collection of information about participants’ real-time experiences in their natural environments (Shiffman et al., 2008). EMA encapsulates many modes of assessment such as transactional diaries (Freedman et al., 2006) or the use of palm-top computers (Shiffman et al., 2008). EMA has the potential to overcome barriers of HRQoL assessment in clinical practice such as time consumption, expensive resources, paper filling and data management (Wright et al., 2003).

The primary benefit of EMA is that real-time experiential measurement circumvents the previously described cognitive biases faced when using retrospective assessment. Experiential variance and fluctuation become informative factors, as EMA seeks to provide a clear picture of subjective experience over the course of time. Indeed, using the electronic beep device *PsyMate*, Maes et al. (2015) administered HRQoL assessment 10 times a day during a 6 days period to both clinical and healthy populations. Their results revealed that real-time reports of moods and symptoms predicted within-person variation in real-time, but not retrospective HRQoL. This finding provides further evidence to suggest that retrospective assessments may provide a biased account of the impact of health problems on the lives of those affected. Moreover, this bias may differ across different conditions. Thus, the EMA promises to provide a valuable improvement to the measurement of HRQoL.

Ecological momentary assessment can also be convenient in clinical practice: remote assessment eliminates time and traveling costs, and allows individuals more flexibility in daily routine (Mehl and Holleran, 2007). The idiographic nature of EMA enables assessment in specific situations. For example, the PedsQL Visual Analog Scale, a momentary HRQoL assessment intended for young children, was found to be reliable for recording their experiences (Sherman et al., 2006).

In light of such potential benefits of the EMA approach, here we provide a protocol that seeks to extend the work of Maes et al. (2015). In particular, we aim to improve the feasibility of the EMA assessment by implementing it in a more accessible device (i.e., mobile phone) and by collecting reports at four time points during a 2-week period aiming to thus minimize respondent’s time-burden. Further, we shall test the psychometric properties and the perceived feasibility of this EMA approach. While some studies employing similar methodologies have reported good ease-of-use and responder satisfaction (Maes et al., 2015), these feasibility analyses have not been comprehensive. Similarly, the validity of developing EMA measures is a key concern: while many EMA studies report their methodology as useful for experiential assessment, few have explicitly validated their measure with direct comparison to traditional measures.

These considerations highlight the need for more evidence on the validity of EMA measures and HRQoL assessed using mHealth applications. Hence, the primary aim of this study is to determine the validity and reliability of using a mHealth application to collect real-time HRQoL. This population is used to identify whether the application is valid in order to determine if there is any merit in testing the new method with a clinical population in the future. This feasibility study aims to test a modality of measuring HRQoL using an established, valid and reliable questionnaire (WHOQOL-BREF). The secondary aims are to investigate individual factors associated with HRQoL variation and to examine the feasibility of this EMA method. These aims will be explored through the following research questions:

1 How do specific domains of HRQoL correlate between real-time and retrospective measurement?
2 Does real-time HRQoL have the same factorial structure as retrospective HRQoL?
3 What is the convergent validity of real-time HRQoL?
4 How reliable is real-time HRQoL, across different time points, compared to retrospective HRQoL?
5 To what extent do mood and current activities account for variation in real-time HRQoL?
6 How do participants judge the feasibility of the mobile application?

In this protocol we provide details about the materials and procedures necessary for EMA of HRQoL using a mobile application. Further, we outline a potential data analysis strategy and prospective discussion of the protocol’s implications and limitations.

## Materials and Equipment

### Literature Search and Choice of Measures

To identify suitable research measures a literature search of electronic databases (PubMed, PsycNet) was performed for literature relevant to HRQoL assessment and mHealth applications. The tools outlined below were selected for their relevance to the research question and their good psychometric properties.

#### Demographic Questionnaire

Participants will be asked for information on gender, occupation (field and level of study, if students), family status, socio-economic status, country of residence, living arrangements, number of children, frequency of smartphone usage, and major life events.

#### HRQoL

The WHOQOL-BREF will be used (The WHOQOL Group, 1994) to assess HRQoL. It contains 26 items comprising four domains: physical health, mental health, social relationships and environment, and two general health items (one for overall quality of life and one for overall health). The instrument has satisfactory validity and reliability in clinical and healthy samples (Lin et al., 2007; Krägeloh et al., 2011). Further, the instrument was developed through a cross-cultural collaboration and its dimensions have been found reliable and valid across many different cultures (Power et al., 1999). This allows for scores obtained in different countries to be combined. For EMA, the wording of the original WHOQOL-BREF questionnaire was modified to be appropriate for real-time responses (e.g., instructing participants to think about their experiences “at this exact moment in time” rather than “over the last 2 weeks”). The original retrospective questionnaire will be used at the end of the 2 weeks and the modified real-time version will be administered during the 2-week assessment. Further, we will only use the physical and mental domains of the WHOQOL-BREF, as the social and environmental domain items were considered less flexible for real-time modification (i.e., people tend not to evaluate social relationships or living conditions on a real-time basis). The questionnaire scoring procedure will be followed. For this study, two domain scores will be provided (physical health and mental health) whilst the two general health items will be scored separately. The mean score of items of each domain will be used for the domain score. Following this, the scores will be converted into a scale for each domain ranging from 0 to 100.

#### Mood and Current Activities

Mood will be assessed in real-time using the Brief Mood Introspection Scale (BMIS; Mayer and Gaschke, 1988) which tests two main components – individuals’ direct experience of specific moods, and the overall “pleasantness” of their mood. The scale has satisfactory reliability and has sufficient sensitivity to distinguish between individuals in low and high mood (Mayer and Gaschke, 1988). The tool will be administered along with the real-time HRQoL questionnaire to assess a potential mood-congruency effect on reports of HRQoL. Participants will be asked to rate their mood on seven mood items (lively, happy, grouchy, sad, tired, nervous, content) on a four-item Likert scale rating from ‘definitely do not feel’ to ‘definitely feel.’ Then, they will be asked to rate their current mood on a scale from -10 to 10 ranging from ‘very unpleasant’ to ‘pleasant.’ The item responses will be summed to obtain a score for each specific mood and total mood score. To further appreciate the context of mood-congruency judgments, participants will also provide information about their current activities prior to reporting their HRQoL.

#### Sleep Quality

Sleep quality will be measured retrospectively at the end of the 2 weeks using the Pittsburgh Sleep Quality Index (PSQI; Buysse et al., 1989). The PSQI asks participants to rate series of items to generate seven component scores: subjective sleep quality, sleep latency, sleep duration, sleep efficiency, sleep disturbance, use of sleep medication and daytime dysfunction. To make the assessment more feasible bed partner ratings will not be recorded. The tool has high test–retest reliability and good validity with both clinical and healthy populations (Backhaus et al., 2002). The association between PSQI and retrospective WHOQOL-BREF scores has been frequently reported (e.g., Meiavia et al., 2013). Here we will investigate whether this relationship is replicated when HRQoL is reported in real-time. If successful we can demonstrate that real-time HRQoL can overcome other biases of retrospective assessment but measuring the same construct.

#### Perceived Stress

At the end of the 2 weeks, participants will complete an online version of the 10-item Perceived Stress Scale (PSS; Cohen et al., 1983; Cohen and Williamson, 1988). The PSS is a widely used questionnaire for measuring the perception of stress. It mainly assesses the unpredictability, uncontrollability and overload of an individual’s life and was designed for use in community samples. The validity and reliability of the scale is well established (Cohen and Williamson, 1988; Roberti et al., 2006). The items request responders to rate how often they experience various feelings and thoughts during the last month on a 5-point Liker scale ranging from *Never* to *Very often*. After reversing the four positively valence items, a sum score is calculated using the 10 items.

#### Social Class

At the end of the 2 weeks, participants will complete an online version of the 10-step ladder social class measurement (Adler et al., 2000), the Sense of Power Scale (*α* = 0.90; Anderson and Galinsky, 2006) and the Sense of Status Scale (*α* = 0.83; Dubois et al., 2015). These measures are employed to assess the relationship between social class and HRQoL, as well as the potential mediating role of social power and/or status. In general, higher socio-economic status is associated with higher HRQoL (Huguet et al., 2008). Such associations will be pursued with the aim to acquire an understanding of the underlying determinants of variation in HRQoL.

#### Feasibility

The Mobile App Rating Scale (MARS; Stoyanov et al., 2015) will be used to assess the feasibility of the mHealth application. The MARS is a multidimensional assessment of mobile application quality, and will be used to reveal both subjective and recurring issues with the app. The MARS has been reliably used by end-users to assess the quality of mHealth apps and it has good internal consistency and test–retest reliability (Stoyanov et al., 2016). For the purposes of the current feasibility evaluation, items not relevant to our application were excluded from the scale (e.g., items about participants’ willingness to pay for the application, as the study has no commercial interest).

## Stepwise Procedure

### Translation Process

Once all research tools have been identified, translation of all materials to six target languages (Danish, German, Greek, Hungarian, Italian, and Spanish; chosen for researcher’s fluency in these languages) was pursued to maximize the accessibility of the mobile application. The WHOQOL-BREF, the PSQI and the PSS have previously been translated and validated in all study languages. Measures that were not available in the study languages (Demographic Questionnaire, Major Life Events [validated version available in German, Hungarian, Italian, and Spanish], the BMIS, Current Activities, Social Class Questionnaires, MARS [validated translation available in Italian]) were translated using the forward–backward translation method and cognitive debriefing (Wild et al., 2005). Within this method, a native speaker of the target language, who was also fluent in English, translated the material into the target language (forward translation). A second native speaker, similarly fluent in English, re-translated the native language translation back to English (backward translation). All discrepancies between the versions were discussed and resolved between the two translators, thereby creating a consensus version of the questionnaire. Finally, the consensus version was administered to two or three native speakers of the target language who were asked to assess its comprehensibility. Any issues raised within this process were brought to the attention of the whole research team and were collectively discussed and resolved. The same translation procedure was followed for the adapted real-time version of the WHOQOL-BREF, which was first devised in English.

### The mHealth Application Development and Data Collection Strategy

**FIGURE 1:**
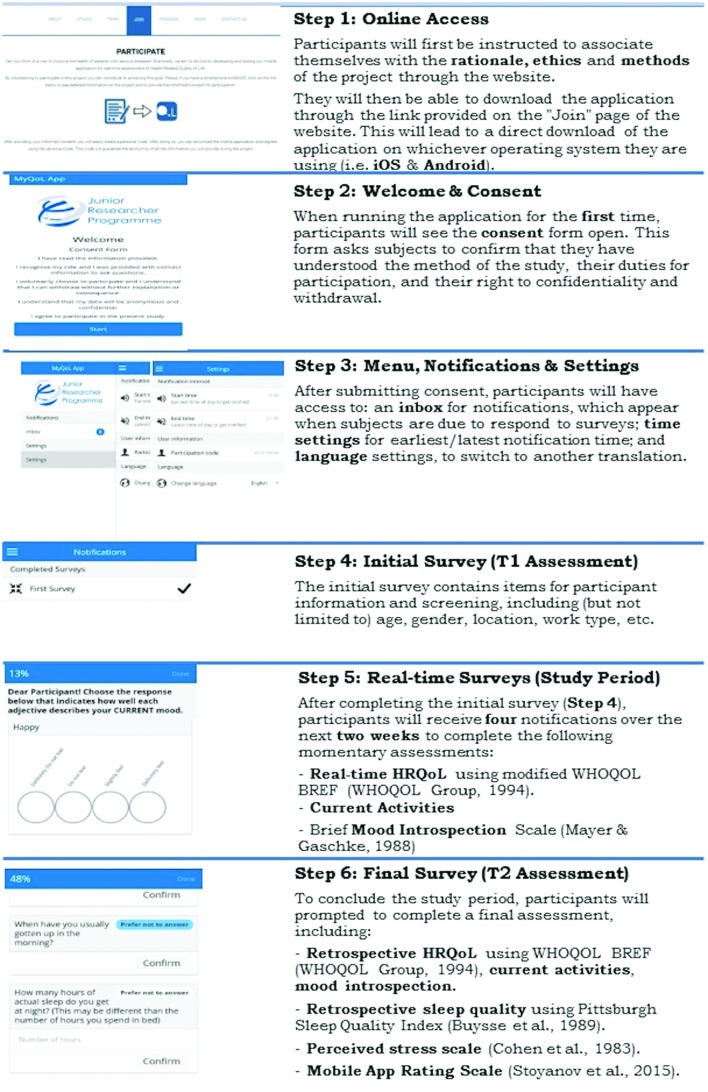
**The assessment process of the study**.

The mHealth application was developed in close collaboration between the researchers and external collaborators with expertise in the development of such software. The researchers had the opportunity to test and provide feedback on early versions of the application. In order to assess areas of improvement the application was piloted with two or three participants in each of the study’s languages. During the study period, participants will be asked to use the mHealth application for a period of 2 weeks, during which the application will send four prompts (at four different time points), asking participants to report their real-time HRQoL, current mood and activities. Prompts will be sent at random times during the day (between an earliest and latest time for notification, defined by participants for their convenience). Participants will be asked to respond within a 6-h interval. Subsequently, at the end of the 2 weeks they will complete a questionnaire assessing retrospective HRQoL, major life events, perceived stress, social class; sleep quality and feasibility (see **Figure 1**).

### Selecting the Target Audience (Participants)

As a means to assess the feasibility of data collection procedure, we aim to recruit 450 healthy participants. Similar sample sizes were used in previous real-time assessment studies (e.g., Maes et al., 2015). This number is more than sufficient for our analysis; an *a priori* power analysis revealed, for example, that only *N* = 46 participants are needed to detect a significant relationship between real-time HRQoL and sleep quality (1 – β = 0.95, α = 0.05, *r* = 0.446). Participants must be over 18 years old and they must own an Android or IOS phone with Internet access. Participants will be excluded if they have a serious mental health condition compromising their ability to respond or their memory. Participants will be recruited through the study’s website, which was designed within a further external collaboration. The link to this website will be distributed via email lists and social media. All participation will be voluntary. On the study website participants will be provided with a web link for downloading the application. The application will contain the study’s Information Sheet and Consent Form. Once Consent is obtained participants will be able to use the mHealth application.

### Proposed Analysis

#### Psychometric Properties of the Real-Time HRQoL Measure

The four real-time HRQoL scores (four time points) will be combined to obtain aggregated real-time HRQoL scores, in order to examine measurement invariance across assessment methods. Measurement invariance will also be examined across time points. Pearson’s correlation coefficient will be calculated between real-time and retrospective domains of HRQoL. The reliability of the real-time and retrospective HRQoL tools will be assessed using Cronbach’s alpha and omega coefficients. Finally, to assess whether the relationship between sleep quality and HRQoL is present when HRQoL is measured in real-time, Pearson’s correlation coefficient will be calculated between the aggregated real-time HRQoL and PSQI score. A two-parameter item response model of real-time HRQoL aggregated data will be used to determine the difficulty and discrimination of questions.

#### Variability in Real-Time HRQoL

Multilevel modeling will be used to obtain estimates of within- and between- person variability in real-time HRQoL. At the first level, coefficients will be estimated for an equation for each person who expresses real-time HRQoL as a function of momentary mood. Subsequently, individual parameters will be used as dependent variables in the level 2 equations to evaluate whether within-person patterns differ across individuals and whether between-person variables (demographics, level of perceived stress, social class, sense of social power and status) and life events might account for the variance.

#### Feasibility

Feasibility will be assessed through analysis of the responses to the MARS questionnaire; percentages will be calculated for the close-ended questions and content analysis will be conducted on responses to the additional open-ended questions.

#### Ethics Statement and Current Status of Project

We went through the typical processes for meeting the ethics requirements for each participating University. We are currently working on finalizing the development of the mobile application and we are planning our pilot study and recruitment procedure.

## Anticipated Results

We expect the project to contribute to the evidence on the validity and reliability of measuring HRQoL using an mHealth application, and to further our knowledge on the development of similar applications. Furthermore, we expect the project to provide insight about the nature of real-time HRQoL data aiming to overcome the cognitive bias and feasibility issues of retrospective assessment. Crucially, there are a number of methodological considerations which merit discussion.

Firstly, the limited assessment time in this study may be problematic. However, we chose this short-time period to minimize missing data and respondent’s time-burden; prolonged assessment periods could exacerbate potential problems with participant commitment and retention. We aim to minimize dropouts by making the application attractive, intuitive and navigable and by allowing participants to skip uncomfortable questions (Reips, 2002). Further, we will engage with participants through the companion website. The study’s website was designed to provide participants with accessible information about the project’s aims. It further contains a step-by-step overview of the participation process, which we expect will aid participants’ engagement. Moreover, the website allows participants to directly contact the research team with arising questions and/or issues. In this way, it further constitutes an important troubleshooting tool.

Another important challenge could be participant compliance; in ecological, natural settings such as the home, it may be expected that participants feel less obliged to provide a complete response within given time-frames. However, promising recent EMA research has shown compliance levels comparable to traditional measures (Mehl and Holleran, 2007; Maes et al., 2015). Participant reactivity is another important consideration and refers to the potential for behavior to be affected by the act of assessing it (Shiffman et al., 2008). Particularly for EMA, one might anticipate frequent prompts to be irritating and impact on response quality. However, studies investigating participant reactivity in EMA have typically found effects to be non-significant (Peters et al., 2000; Stone et al., 2003). Crucially, these concerns are recognized and they shall be evaluated as part of the planned feasibility analysis.

Finally, since this is a feasibility study no power calculation or methods of Limits of Detection (LOD) or Limits of Quantification (LOQ) were established as we view the feasibility findings as the avenue to establish these for future studies and larger trials. Such subsequent investigations would further allow validating the translations that were prepared for the current study. Whilst we employed a rigorous translation procedure, a larger sample size would allow an estimate of measurement invariance across language versions.

Overall, we anticipate that our results will elucidate the relevance of such potential limitations. Further, we consider that our results, along with the current protocol, will aid future research exploring the potential of using EMA of HRQoL with a mobile application in clinical settings. Routine assessment of HRQoL in such settings is known to benefit patient-physician communication (Velikova et al., 2004), may facilitate clinical assessment and can be crucial for monitoring individual HRQoL over course of treatment. However, such assessments are also associated with substantial expenses and patient’s time-burden. Future cost-effectiveness analyses can shed a light in this issue but we consider that EMA delivery in a mobile application may hold the potential to overcome these feasibility drawbacks.

## Author Contributions

This study was conceived and initially designed by APK. All of the authors further contributed to the research design, methodology, analysis plan and prospective discussion. First authors SM and DT drafted the first manuscript and were assisted by PM, VEM, CVO, BS, TW, and APK who contributed with additional writing and critical commentary. All authors approved the final manuscript.

## Conflict of Interest Statement

The authors declare that the research was conducted in the absence of any commercial or financial relationships that could be construed as a potential conflict of interest.
